# Optical fibre-based quantum random number generator: stochastic modelling and measurements

**DOI:** 10.1038/s41598-025-95414-y

**Published:** 2025-03-29

**Authors:** Michał Dudek, Grzegorz Siudem, Grzegorz Kwaśnik, Wojciech Żołnowski, Marek T. Życzkowski

**Affiliations:** 1 Institute of Applied Physics, Military University of Technology, 00908 Warsaw, Poland; 2 Faculty of Physics, Warsaw University of Technology, 00662 Warsaw, Poland; 3 Institute of Optoelectronics, Military University of Technology, 00908 Warsaw, Poland; 4 Department of Complex Systems, National Center for Nuclear Research, 05400 Otwock, Poland

**Keywords:** Fibre optics and optical communications, Computational science

## Abstract

In this work, we present a study of a quantum random number generation system based on a branching path approach with spatial superposition principle, realised using fibre optics. The analysis of the experimental system was supported by the development of a stochastic model of the entropy source, which, to the best of the authors’ knowledge, has not yet been properly described. This resulted in the analytical equations for the probability of possible output quantum states depending on the initial properties of the system. Based on the presented model, the quantum efficiency and the Shannon entropy were calculated and compared with experimentally obtained values, which resulted in full agreement between these data. Additional statistical tests were performed on random numbers obtained theoretically and experimentally to confirm their high degree of randomness and their usability in cryptographic applications. The fact that the developed system is based on fibre optics allows it to operate in stable conditions with a final efficiency at the level of 15%, which provides a random number generation rate of about 8 kb/s. The developed system is used as an input to the quantum key distribution system, which has possible applications in cryptography or military and commercial secure communications.

## Introduction

Random numbers are numbers that cannot be predicted and are generated by a random process. Their role in modern civilisation is not only limited to games and lotteries, but they can also be found in most aspects of our daily lives, even if we are not aware of it. The generation of such random numbers is crucial in many applications, including science^1^, lotteries^2^, secure communication and cryptography^3,4^ or simulations^5,6^. One of the most notable examples of the application of random numbers is quantum key distribution (QKD)^7^, which is where the modern cryptography meets communication. QKD systems provide a way for distribution of a secure key used for further communication between at least two parties. The key itself must be sufficiently complex and random to be viable for this purpose, and the exchange of this key usually involves highly secure means of communication utilizing either Heisenberg uncertainty principle^8^ or quantum entanglement^9^. Therefore, the main source of randomness (or entropy) for this kind of systems comes directly from random number generators (RNGs).

Randomness has many definitions and there are many ways to verify it. In this article, we follow an approach developed by A. Kolmogorov^10^, according to which an output string appears random if it has a high Kolmogorov complexity^11^. Later, many other statistical tests were developed and introduced for general use, like, e.g., the NIST Statistical Test Suite^12^, *TestU01*^13^ or *dieharder*^14^. However, in practice, obtaining a true random number sequence is not a trivial matter.

True random number generators (TRNGs) are the solution for avoiding predictive string sequences, which may occur in case of pseudorandom number generators^15^ based on pure algorithmic generation of such numbers. In this solution, the output string is associated only with the unpredictable physical phenomenon^16^, which guarantees high randomness of the obtained data. The entropy generation can be based on different physical phenomena, such as, e.g., electrical noise or radioactive decay^17^. Therefore, there must not be any analytical relation or algorithm that describes the realization of this source, e.g., time courses^18^.

Among the TRNGs, we distinguish several subgroups, based on various parameters of the generators^2^. The main subgroups are noise-based random number generators, free running oscillator random number generators, chaos random number generators, and quantum random number generators (QRNGs). In QRNGs, the random number generation is based on a quantum mechanics principle^19^. Therefore, the outcome cannot be predicted before measurement^20^. Most notable hardware implementations of QRNG systems are based on radioactive decay^16^, noise^21^, and different implementations of optical phenomena, e.g., branching path generators^22^, time of arrival generators^23^, photon counting generators^24,25^, generators based on Raman scattering^26^ or transition effect ring oscillator^27,28^, and many others^29,30^. They differ not only in the physical phenomena used for random number generation, but also in reliability and speed of data generation. So far, most of the published QRNG solutions were focused mainly on the experimental implementation of the system and output randomness analysis without providing theoretical description in a form of stochastic model.

The most basic optical QRNG configuration, called branching path generator, registers photons downstream of the beam splitter^20^. This setup includes a single-photon source, a beam splitting element, and two single-photon detectors. Single photons can be generated by a specific source, which emits single photons on-demand or as a result of suppressing a multi-photon laser beam to single photons. Each solution outcome should be a single photon transmitted to the beam splitter. Common beam splitters are theoretically 50/50, which gives a 50% chance of transmitting the photon into each path after the beam splitter^31^. After passing through a beam splitter, a photon is detected by one of the single-photon detectors. The event of photon detection by only one of the detectors is therefore considered as a random output bit 1 or 0 - depending on which detector registered a photon. The branching path QRNGs based on spatial superposition can be realised either in free-space optics^22,32–34^ or by using fibre optics^35^. Although this solution provides a high level of safety and high randomness in the sequence, it does not guarantee a high speed of bit generation. In this type of QRNG, the generation rate is limited by the parameters of the source and the physical limitations of the detectors.

To ensure the reliability of QRNG devices, it is necessary to comply with the established safety standards. The two most important standards that validate QRNGs are BSI AIS 20/31^36^ and NIST SP 800-90B^37^. One of the requirements for proper validation of QRNGs is the development of a stochastic model of the entropy source. Although the idea of described RNG is well known, a corresponding stochastic model has not yet been properly described to the best of our knowledge, and the generic model proposed in^38,39^ is not suitable in this case. Therefore, in this article, we present a laboratory QRNG system based on the branching path principle, and a full analytical description of the stochastic model developed for this system, along with a comparison of the experimental results with the theoretical ones.

## Materials and methods

### Experimental setup

**Fig. 1:**
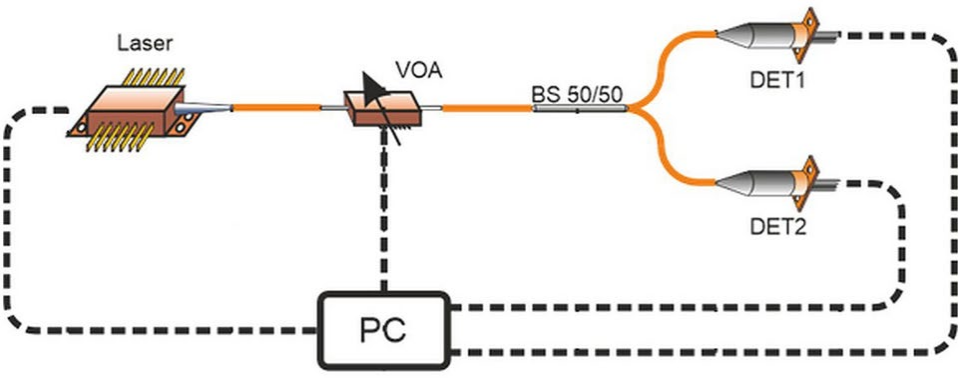
Experimental setup scheme.

The developed system is a practical implementation of a QRNG setup based on the idea of a single-photon detection after a beam-splitting element. As a result, each iteration of the experiment produces a single random bit. The experiment is repeated until the desired number of bits is reached. The scheme of the setup is presented in Fig. 1. The laboratory generator system consisted of the following components:

- A 640 nm pulsed laser PicoQuant CPDL-S-640 (Laser),
- Variable optical fibre attenuator Thorlabs V600A (VOA),
- A 50/50 fibre optic splitter Thorlabs TW 630R5A1 (BS 50/50),
- Two single-photon detectors Laser Components COUNT-10C-FC (DET1 and DET2),
- ARTIQ Sinara and user computer - control and readout system (PC).

**Fig. 2:**
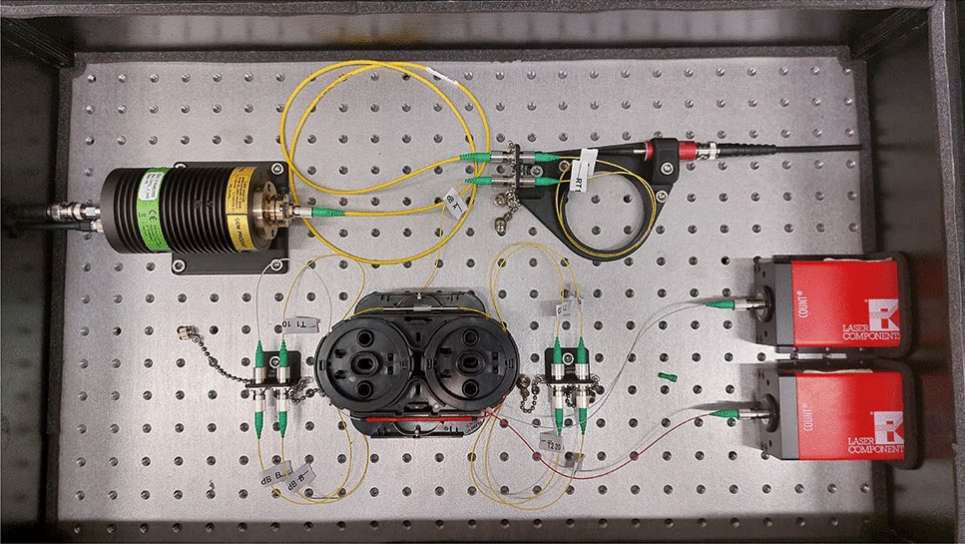
Photo of the optical part of the QRNG system.

All optical fibres and other components were equipped with FC/APC connectors to minimise reflections. Additionally, to improve the range of attenuation in the variable optical fibre attenuator, a set of constant optical fibre attenuators (two Fibrain 10 dB attenuators and two Fibrain 20 dB attenuators) were placed in the system right before the fibre optic splitter. Prior to construction, all optical elements were thoroughly characterised using standard procedures developed for fibre optic components^40^. A photo of the optical part of the QRNG system is shown in Fig. 2.

#### Light source and optical attenuators

The photon source in the system was a 640 nm pulsed laser. The laser can operate at any frequency between 0 and 40 MHz and was triggered by a rising edge of an external pulse. To fulfil the assumption of quantum number generation, the laser signal was attenuated to the level of single photons. This was done by using a set of optical fibre attenuators. The attenuators have been selected to provide an expected high level of attenuation and the ability to adjust attenuation values. The measured attenuation of constant attenuators was 60.88 dB, whereas the VOA provided additional attenuation from 2.5 dB up to above 30 dB. During the experiment, the laser was triggered by the control unit and then an attenuated laser pulse was passed to the beam-splitting element.

#### Beam splitter

The beam-splitting element is the fibre coupler. It divides the signal coming from the laser into two independent paths according to the proportions specified in the specifications. To divide the signal into two paths with equal probability, a 50/50 coupler is used. The probabilities of the photon passing to each output are theoretically equal. The outputs of the coupler are connected by optical fibres to single-photon detectors. A generated single photon can “choose” randomly only one of the paths and is passed to the detector. In the experimental setup the beam splitting ratio was measured to be $-3.43$ dB/$-3.28$ dB ($45.39\%$/$46.99\%$) with optical loss equal to $-11.18$ dB ($7.62\%$).

#### Detectors

Single-photon detectors are installed at the end of each arm of the coupler. The detectors operate in gating mode to improve the signal-to-noise ratio. The gate is operated by an external trigger source correlated with the pulsed laser. The gate is open only for the expected photon arrival time. In the case of detection, an output signal is generated. These signals are counted by the control unit. The expected states are 00, 01, 10 or 11. Registered values are stored in the control unit buffer. The dataset containing these bits is considered raw data before postprocessing. The detectors used in the experimental setup are characterised by photon detection efficiency equal to 54.14% for DET1 and 52.24% for DET2, dark count rate equal to 6.3 $\hbox {s}^{-1}$ for DET1 and 8.7 $\hbox {s}^{-1}$ for DET2, and afterpulsing equal to 0.04% for DET1 and 0.03% for DET2.

#### Control and readout system

The control unit is responsible for handling the experiment and communicating with the user’s PC. During the experiment, the unit synchronises events and stores data. Events that have to be synchronised are pulse generation, detector gate opening and closing, and recording detector response. Due to the need to operate in the nanosecond regime, the ARTIQ Sinara system was used. All devices used in the system have a transistor-transistor logic (TTL) standard interface. The Sinara controller is equipped with Kasli and digital input/output (DIO) cards:

- Kasli module—constitutes the platform controller. The module is controlled from the control computer and then communicates with other modules installed in the platform.
- DIO-TTL—having 8 digital inputs-outputs in the TTL standard, this module was used to communicate directly with the components of the quantum system, such as the laser and detectors. This module is responsible for handling all input/output signals.

### Experiment description

#### Measurement and raw data generation

Before starting the experiment, the user has to enter the expected length of the dataset of random bits. Based on this input variable, the number of iterations of the experiment is determined. A single cycle of the experiment includes photon generation, selection of the optical path, and photon detection.

During the experiment, the control unit communicates with subsystems via TTL outputs/inputs. A strictly timed sequence of pulses is generated. At the same time, the counting of input pulses on inputs dedicated to detectors begins. After the completion of a single cycle, the counts are stored in the device memory. The cycle is then repeated until the preset number of iterations is reached. Before the postprocessing step, count streams are transformed into single bitstream. This stream is the raw data output of the generator and the input to the postprocessing.

A single cycle, consisting of providing output control signals and registering input from the detectors, took 133 ns, which secured the signal processing rate of approximately 7.5 MHz. After that the registered counts had to be saved in the memory by appending them to existing streams, which was about 100-times slower than the measurement cycle. Therefore, owing to the limitations of the system configuration, the final maximum detection frequency was set to be approximately 50 kHz, which greatly limited the bandwidth of the generated random numbers. Further increase in the bandwidth may be achieved by using custom dedicated electronic devices, which was not the scope of this study.

#### Postprocessing

As the resulting measurement is affected by noise and imperfections in the used components, it is necessary to introduce a postprocessing step in the generation process. The purpose of this procedure is to obtain true randomness from the raw measurement data. This can be accomplished via the implemented algorithms or machine of states in the generator^41^. The inputs for that algorithm are bitstreams captured during the experiment. The next step, depending on the implemented algorithm, is to create an output bitstream based on analysis of the input. The goal of this process is to decrease the influence of the environment or the inequality of power distribution through fibre optic connectors. After postprocessing, states 00 and 11 are removed from the bitstream and then every consecutive bit value is chosen from the alternating detector (i.e., DET1, DET2, DET1, DET2,...). The sequence thus obtained is the generator random output sequence and is passed to the user.

#### Summarised workflow

The operating principle of the developed QRNG system can be summarised as follows:

1. Set initial parameters—mostly the size of the output dataset to be generated;
2. Device calibration—setting up the attenuation and determining the quantum efficiency;
3. Start-up test—ensuring the functionality of the system when it is started;
4. Random bits generation followed by their storage in the device buffer;
5. Total failure test and online test followed by Shannon entropy calculation and estimation of final efficiency—performed on the buffered dataset;
6. If all tests are passed, save the data into output files together with the statistics info;
7. Further statistical tests based on BSI AIS 20/31^36^ or NIST SP 800-90B^37^.

To prove a true randomness of the generated raw random numbers, each RNG should pass the validation process. One way is to fulfil the requirements of one of the aforementioned international norms (BSI AIS 20/31 or NIST SP 800-90B). In our study, we prepared a stochastic model of the entropy source and performed an additional set of statistical tests, based on the NIST Statistical Test Suite^12^.

### Stochastic model of the entropy source

#### Initial problem description

The stochastic model was designed based on several initial assumptions listed below.

- Laser pulses attenuated to single photons—the laser-generated pulses are attenuated to a level where the average number of photons per pulse follows the Poisson distribution.
- The power splitting ratio at the fibre splitter is not in perfect 50/50 proportions and thus the transmission probabilities to both detectors are not equal.
- Differences in detection probabilities between two different detectors (production spread)—the quantum efficiency of each detector is slightly different.
- Dark count—detectors may count false detections due to thermal noise.
- Afterpulsing phenomenon—when a photon is detected on a detector, the probability of another false detection increases because of charge accumulation inside the diode.

An additional assumption not included in the final stochastic model of the entropy source was the crosstalk between detectors. As a result of electrical coupling in a control and recording device, a detection on one detector may cause a detection on another detector because of charge transfer. However, this phenomenon was not identified in any way in the experimental system. The measurements and thorough signal analyses indicated that this phenomenon either did not occur in our system due to the proper separation of both detection channels or, if it did occur, its influence on the whole process was negligible.

#### Probability formulation

Based on the presented description, it was possible to formulate the probability of obtaining any state simultaneously on both detectors $(\sigma _1,\sigma _2)\in \{(0,0),(0,1),(1,0),(1,1)\}$ at time *t*, as presented below:

$$\begin{aligned} {\mathbb {P}}&\left[ \Sigma _t=(\sigma _1,\sigma _2) \right] = e^{-\lambda \left[ (p_1-s_1)(q_1+1)+(p_2-s_2)(q_2+1)\right] }(-1)^{\sigma _1+\sigma _2}\times \\&\times \left[ (1-r_1) e^{\lambda (p_1-s_1) q_1}+\sigma _1(-1)^{\sigma _1} e^{\lambda (p_1-s_1) (q_1+1)}+e^{\lambda (p_1-s_1)}\right] \times \\&\times \left[ (1-r_2) e^{\lambda (p_2-s_2) q_2 }+\sigma _2 (-1)^{\sigma _2} e^{\lambda (p_2-s_2) (q_2+1)}+e^{\lambda (p_2-s_2)}\right] . \end{aligned}$$

The final time-dependent probabilities of each possible state on both detectors ($\pi _{00}$, $\pi _{01}$, $\pi _{10}$ and $\pi _{11}$) can be expressed as:

$$\begin{aligned} \pi _{00}=&C \left[ e^{\lambda (p_1-s_1)}+\left( r_1-\Delta _1-1\right) e^{\lambda (p_1-s_1) \left( q_1+\Delta _1\right) }\right] \left[ e^{\lambda (p_2-s_2)}+\left( r_2-\Delta _2-1\right) e^{\lambda (p_2-s_2) \left( q_2+\Delta _2\right) }\right] , \\ \pi _{01}=&C e^{\lambda \left[ \Delta _2 (p_2-s_2)+p_1-s_1\right] } \left[ e^{\lambda (p_2-s_2)}-e^{\lambda (p_2-s_2) \left( q_2+1\right) }+\left( r_2-1\right) e^{\lambda (p_2-s_2) q_2}\right] \times \\ &\times \left[ \left( -r_1+\Delta _1+1\right) e^{\lambda (p_1-s_1) \left( q_1+\Delta _1-1\right) }-1\right] ,\\ \pi _{10}=&C e^{\lambda \left[ \Delta _1 (p_1-s_1)+p_2-s_2\right] } \left[ e^{\lambda (p_1-s_1)}-e^{\lambda (p_1-s_1) \left( q_1+1\right) }+\left( r_1-1\right) e^{\lambda (p_1-s_1) q_1}\right] \times \\ &\times \left[ \left( -r_2+\Delta _2+1\right) e^{\lambda (p_2-s_2) \left( q_2+\Delta _2-1\right) }-1\right] ,\\ \pi _{11}=&C e^{\lambda \left[ \Delta _1 (p_1-s_1)+\Delta _2 (p_2-s_2)\right] } \left[ e^{\lambda (p_1-s_1)}-e^{\lambda (p_1-s_1) \left( q_1+1\right) }+\left( r_1-1\right) e^{\lambda (p_1-s_1) q_1}\right] \times \\&\left[ e^{\lambda (p_2-s_2)}-e^{\lambda (p_2-s_2) \left( q_2+1\right) }+\left( r_2-1\right) e^{\lambda (p_2-s_2) q_2}\right] ,\\ C=&\left[ -e^{\left( \Delta _1+1\right) \lambda (p_1-s_1)}+e^{\lambda (p_1-s_1)}+\Delta _1 \left( -e^{\lambda (p_1-s_1) \left( q_1+\Delta _1\right) }\right) +e^{\lambda (p_1-s_1) \left( q_1+\Delta _1+1\right) }\right] ^{-1}\times \\&\left[ -e^{\left( \Delta _2+1\right) \lambda (p_2-s_2)}+e^{\lambda (p_2-s_2)}+\Delta _2 \left( -e^{\lambda (p_2-s_2) \left( q_2+\Delta _2\right) }\right) +e^{\lambda (p_2-s_2) \left( q_2+\Delta _2+1\right) }\right] ^{-1}, \end{aligned}$$

where $\lambda$ is the expected value of the photons in the system (based on the initial attenuation), $p_i$ is the probability of obtaining a photon in the *i*-th path (based on splitting ratio), $s_i$ is the probability that the photon was lost in the *i*-th path (attenuated or reflected), $q_i$ is the probability that a photon was detected by the *i*-th detector when there was photon to be detected (i.e., photon detection efficiency - true positive), $r_i$ is the probability that no photons were detected by the *i*-th detector when there were no photons to be detected (true negative), and $\Delta _i$ is the probability of afterpulsing for the *i*-th detector. The exact derivation of the equations above is presented in the Supplementary materials 1.

#### Final parameters determination

Based on the state of detectors at time *t*, it is possible to generate a bit of information through a von Neumann procedure - if both detectors registered the same state (either 00 or 11) then the result is discarded, otherwise the bit is set to the value of the first detector (meaning 0 in the case of state 01 and 1 in the case of state 10). Therefore, the probability of successfully obtaining either state 01 or 10 (meaning that only one of detectors properly registers a photon) can be written as:

$$\begin{aligned} P=\pi _{01}+\pi _{10}. \end{aligned}$$

This value may also be considered as the efficiency of the bit generation process.

The Shannon entropy from the desired states is calculated as:

$$\begin{aligned} H=&-\left[ \frac{\pi _{01}}{\pi _{01}+\pi _{10}}\log _2\left( \frac{\pi _{01}}{\pi _{01}+\pi _{10}}\right) +\frac{\pi _{10}}{\pi _{01}+\pi _{10}}\log _2\left( \frac{\pi _{10}}{\pi _{01}+\pi _{10}}\right) \right] =\\ =&\log _2(\pi _{01}+\pi _{10})-\frac{\pi _{01}\log _2(\pi _{01})+\pi _{10}\log _2(\pi _{10})}{\pi _{01}+\pi _{10}}. \end{aligned}$$

It can be expressed in bits per bit or bits per byte after multiplication of the result by 8.

To validate the applicability of the developed model to the experimental setup, we used the coefficient of determination $R^2$ (goodness of fit) to compare the experimental results with those obtained numerically. It can be calculated as:

$$\begin{aligned} R^2=1-\frac{\sum _{i}{\left( y_i-f_i\right) ^2}}{\sum _{i}{\left( y_i-{\overline{y}}\right) ^2}}, \end{aligned}$$

where $y_i$ represents the values in the experimental dataset, ${\overline{y}}$ represents the mean value of the experimental dataset, and $f_i$ represents the values in the modelled dataset. The numerator in this equation is the sum of squares of residuals, whereas the denominator is the total sum of squares.

### Statistical tests

The generated random sequences were tested according to the NIST SP800-22^12^ standard and to check their randomness. The NIST Statistical Test Suite is a toolkit containing 15 tests designed to assess the randomness of binary sequences, regardless of their length and source. The tests are designed to detect various forms of nonrandomness that may be present in a sequence.

Each statistical test verifies the null hypothesis (H0), which assumes that the sequence under investigation is random. The alternative hypothesis (Ha) states that the sequence is not random. A statistical test leads to the acceptance or rejection of H0 based on a comparison of the value of the test statistic with a critical value determined from a reference distribution. The critical value is usually set at 99% and a test statistic is calculated for the resulting sequence. If the test statistic exceeds the critical value, H0 is rejected.

Hypothesis testing can lead to two types of errors: type I (false rejection of H0) and type II (false acceptance of H0). The probability of a type I error is the significance level of the test, which is usually 0.01%. The probability of a type II error is more difficult to estimate because of the variety of possible nonrandomness.

## Results

The results obtained during the measurements were compared with those from the numerical modelling.

The experimental setup parameters are summarised in Table 1, where $p_1$ and $p_2$ are the probabilities of obtaining a photon in either path (based on splitting ratio), $q_1$ and $q_2$ are the probabilities that a photon was detected by either detector when there was photon to be detected (i.e., photon detection efficiency - true positive), $r_1$ and $r_2$ are the probabilities that no photons were detected by either detector when there were no photons to be detected (true negative), which are based on the dark count rate and detection frequency, and $\Delta _1$ and $\Delta _2$ are the probabilities of afterpulsing for each detector.

**Table 1:** Experimental setup parameters.

Parameters	Values
$p_1$ (measured)	45.39% (−3.43 dB)
$p_2$ (measured)	46.99% (−3.28 dB)
$q_1$ (measured)	54.14%
$q_2$ (measured)	52.24%
$r_1$ / dark count rate	99.979% / 6.3 $\hbox {s}^{-1}$
$r_2$ / dark count rate	99.971% / 8.7 $\hbox {s}^{-1}$
Detection frequency	50 kHz
$\Delta _1$	0.04%
$\Delta _2$	0.03%

### Entropy determination

To obtain a full characterization of the developed QRNG, we tested it for different attenuation (expected number of photons in the system) $\lambda$ from 0.1 to 7 photons. In this whole range, the Shannon entropy for datasets of 10 Mb after postprocessing was always greater than 7.99999 bits per byte (0.99999 bits per bit), whereas the raw entropy (before postprocessing) was greater than 7.99 bits per byte (0.999 bits per bit) for attenuation from 0.2 to 7 photons and 7.98 bits per byte (0.9975 bits per bit) for attenuation of 0.1 photons. The parameter that mostly influenced the lowered entropy for high attenuation (below 0.5 photons) was the detection frequency due to the increased influence of the dark count rate of the detectors, which is constant in time. Hence, the detection frequency should be higher than approximately 30 kHz, while at the same time, the maximum possible detection frequency of the developed system was about 50 kHz.

### Optical loss confirmation

**Fig. 3:**
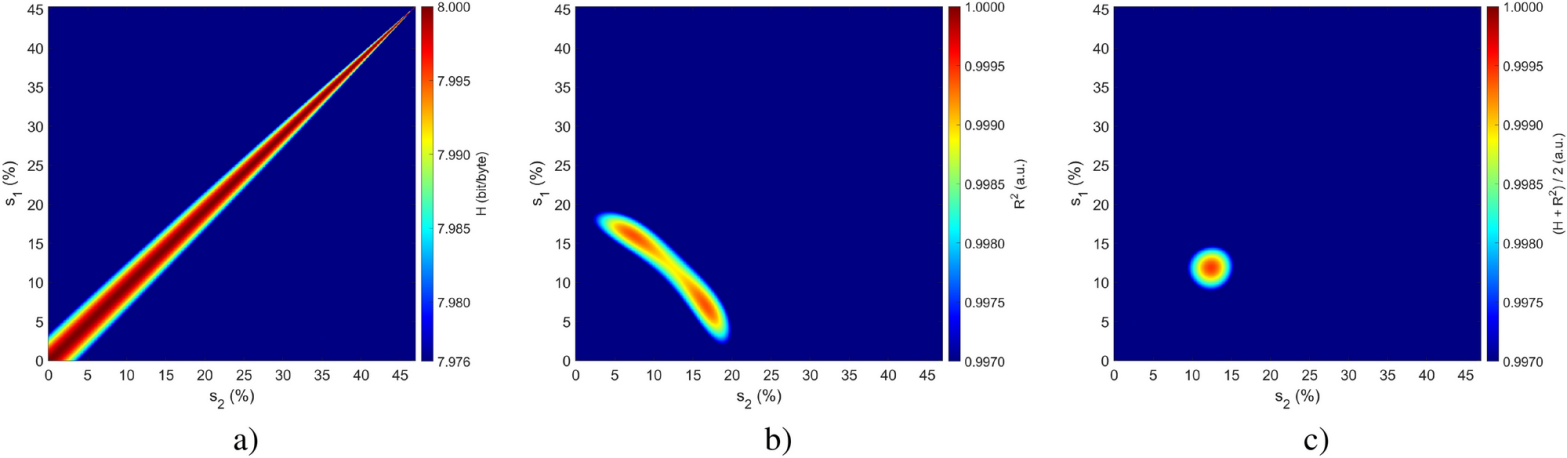
Distributions of (**a**) the mean entropy value *H*, (**b**) the goodness of fit value $R^2$, and (**c**) both of these values combined in terms of loss values $s_1$ and $s_2$.

For all of the parameters, only losses $s_i$ were not known beforehand due to the inability to measure the exact losses of the optical fibre connections in both paths after the connections were made. Therefore, they had to be empirically established based on the comparison between results obtained numerically and those obtained experimentally. In order to do that, we calculated for possible pairs of $s_1$ and $s_2$, both the mean entropy value *H* for the expected number of photons in the system $\lambda$ from 0.1 to 7 photons as shown in Fig. 3a, and the goodness of fit $R^2$ between the experimental and modelled probability of success *P* (obtaining either state 01 or 10), as shown in Fig. 3b. The combined parameters yield the distribution presented in Fig. 3c, in which the maximum value corresponds to the highest value of entropy with the corresponding highest value of the goodness of fit. Therefore, we obtained a pair of estimated $s_1$ and $s_2$ values, which were equal to 11.9% ($-9.24$ dB) and 12.4% ($-9.07$ dB), respectively.

### Stochastic model validation

**Fig. 4:**
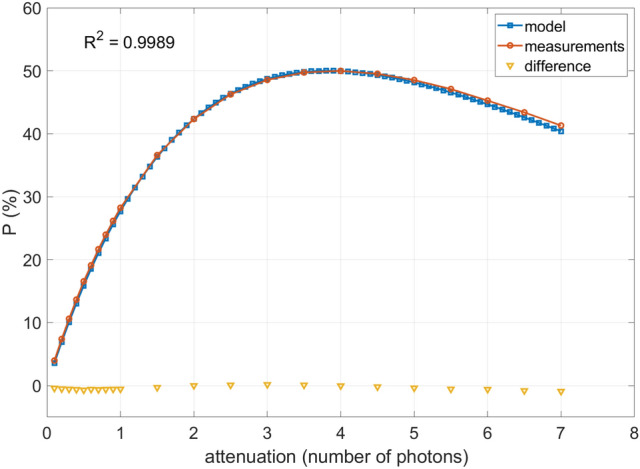
Comparison between experimental data and modelled data for previously estimated loss values $s_1=11.9\%$ and $s_2=12.4\%$.

Based on the obtained estimated values of losses in both channels, it was possible to calculate theoretical Shannon entropy of the system and also compare the probability of success *P* obtained experimentally and numerically, which is presented in Fig. 4 together with the difference between these two curves. The goodness of fit equal to 0.9989 confirms that the developed model is in good agreement with the experimental results and may be used to predict the outcomes. The theoretical entropy for a detection frequency of 50 kHz was determined to be greater than 7.99999 bits per byte (0.99999 bits per bit) in the whole range of attenuation, which is also in good agreement with the value obtained experimentally.

The highest efficiency of the developed QRNG system was close to 50%, which was achieved for the attenuation of approximately 4 photons in the system. In this case, the highest probability of obtaining states 01 and 10 did not correspond with the high randomness of the produced datasets because of the increased number of photons in the system. For the process to remain quantum and provide truly random results, the expected number of photons in the system should be less than 1 photon. This is the reason why not only entropy and quantum efficiency should be taken into account, but also a set of statistical tests needs to be performed on the output datasets to ensure their randomness.

What is also evident from graph in Fig. 4 is that the efficiency of bit generation is almost directly proportional to the expected number of photons in the system when this value is less than 1. The efficiency for the number of photons in the system equal to 1 is below 30% and drops down towards lower values. Therefore, it can be concluded that about 70-90% of the measurements in this range are discarded due to the quantum nature of the experiment, which greatly affects the final random number generation rate.

### Statistical tests

The most common set of statistical tests is provided in the NIST Statistical Test Suite^12^, which was performed both for experimental and numerically obtained data, as summarised in Table 2 and discussed below.

**Table 2:** Statistical test results for experimental data with 1 Mb, where P—success and X—failure.

No.	Test	0.1	0.2	0.3	0.4	0.5	0.6	0.7	0.8	0.9	1.0
1	Frequency (monobit) test	P	P	P	P	P	P	P	P	P	P
2	Frequency test within a block	P	P	P	P	P	P	P	P	P	P
3	Runs test	P	P	P	P	X	X	X	X	X	X
4	Test for the longest-run-of-ones in a block	P	P	P	P	P	P	P	P	P	P
5	Binary matrix rank test	P	P	P	P	P	P	P	P	P	P
6	Discrete Fourier transform (spectral) test	P	P	P	P	P	P	P	P	P	P
7	Non-overlapping template matching test	P	P	P	P	P	P	P	P	P	P
8	Overlapping template matching test	P	P	P	P	P	P	P	P	P	P
9	Maurer’s “Universal Statistical” test	P	P	P	P	P	P	P	P	P	P
10	Linear complexity test	P	P	P	P	P	P	P	P	P	P
11	Serial test	P	P	P	P	P	P	P	P	P	P
12	Approximate entropy test	P	P	P	P	P	P	P	P	P	P
13	Cumulative sums (cumsums) test	P	P	P	P	P	P	P	P	P	P
14	Random excursions test	P	P	P	P	P	P	P	P	P	P
15	Random excursions variant test	P	P	P	P	P	P	P	P	P	P

We performed the set of tests 10 times on experimentally generated bitstreams with a length of 1 Mb. A test was considered to be successfully passed if all of 10 repetitions were passed. If at least one repetition did not pass the test, it was considered as failed. As shown in Table 2, it is evident that above 0.4 expected number of photons in the system, the generated output datasets did not pass the runs test and had to be discarded. The runs test allows to determine whether the number of runs of ones and zeros of various lengths is as expected for a random sequence. If the oscillation between such zeros and ones is too fast or too slow, the test is considered as failed, which happened in the cases from 0.5 up to 1.0 photons in the system, with number of fails increasing together with the expected number of photons in the system.

The set of statistical tests was also performed on datasets obtained with the developed stochastic model based on input pseudorandom numbers. The output bitstreams had a length of 100 Mb and were obtained for the same values of expected number of photons in the system as for experimental data (0.1-1.0). All of the tests were passed each time. Therefore, the only limitation on the randomness of the generated datasets originates directly in the experimental system.

Based on the presented comparison of statistical tests, it is evident that for the developed QRNG system to provide true random output data, the attenuation should be in the range of 0.3-0.4 photons in the system. This provides an efficiency of approximately 15% and the final random number generation speed at the level of 1 kB/s (8 kb/s).

## Discussion

We developed a complete optical fibre-based QRNG system together with its stochastic model, which is thoroughly described in the Supplementary materials 1, and provided the analytical equations for the final probabilities of each possible quantum state (00, 01, 10, 11) depending on the system properties. To the best of our knowledge, such model for a branching path generator has not been reported before. Based on the comparison of experimental and modelled results it was possible to confirm the usability of the presented QRNG system and determine its functional parameters. The stochastic model presented in this work by means of the probability evolution analysis allows to perform a proper analysis of the QRNG system and determine the influence of its initial properties on the entropy of the output random numbers.

The final Shannon entropy values obtained for both the model and experimental datasets were greater than 7.99999 bits per byte (0.99999 bits per bit) for the whole analysed attenuation range, which is much more than the 7.976 bits per byte (0.997 bits per bit) required by the BSI AIS 20/31^36^. All the statistical tests performed both on the experimental and numerically obtained data confirmed the randomness of the produced datasets and determined that the expected number of photons in the system should be at the level of 0.3–0.4 photons for the output data to be truly random. Therefore, the final efficiency of the system (probability of obtaining a proper random bit) is at the level of 15%, which gives a bandwidth of approximately 1 kB/s (8 kb/s) due to the detection frequency limitations originating from multi-purpose Sinara controller. The bandwidth can be increased by using fast dedicated controller with sampling rate >100 MHz, which was beyond the scope of the present study. Nevertheless, the generation rate of our system can be compared with other non-commercial branching path QRNGs, as summarised in Table 3. For all reported QRNGs with additional statistical tests performed on the output numbers, the bandwidth was at the level of 10-30 kb/s. Commercial QRNGs based on branching path approach can achieve random bit rate at the level of 4 Mb/s^42^.

**Table 3:** Comparison with other branching path QRNGs. *Authors performed only three simple tests: calculation of the mean value of 8-bit blocks, calculation of the entropy for 8-bit blocks, and the Monte Carlo estimation of $\pi$.

Source	Bandwidth	Remarks
Jennewein et al.^22^	1 Mb/s	no statistical tests performed*
Wang et al.^32^	100 kb/s	no statistical tests performed
Soubusta et al.^35^	28 kb/s	based on *diehard* statistical tests
Bronner et al.^33^	10 kb/s	based on NIST statistical tests
Xu et al.^34^	10 kb/s	based on NIST statistical tests
This work	8 kb/s	based on NIST statistical tests

The main purpose of the developed QRNG system is its application as an input to the QKD system. Random numbers generated by the presented entropy source are in the next step encrypted using the Advanced Encryption Standard (AES) and passed to the QKD system in order to provide random and secure key for further communication. The main goal of using random numbers in such systems is to ensure the secrecy of the data, since it is not possible to determine past and future bitstream values based on the current ones (forward and backward secrecy), and this is what the presented QRNG system realises. Utilization of the AES algorithm additionally improves this property and enhances security. QRNG and QKD systems are widely used in cryptography, banking, lottery and gambling, military and other applications where high randomness and secure communication are crucial.

## Supplementary Information

Supplementary Information 1.

## Data Availability

The data supporting the findings of this study are available from the corresponding author upon reasonable request.
