# Supplementary material for: Optical fibre-based quantum random number generator: stochastic modelling and measurements
Source: Sci Rep. 2025 Mar 29;15:10849. doi: 10.1038/s41598-025-95414-y (PMC11954864; doi:10.1038/s41598-025-95414-y)
Supplement: Supplementary file 1 — Supplementary Information 1. [file 41598_2025_95414_MOESM1_ESM.pdf]

Optical fibre-based quantum random number generator — stochastic modelling and measurements – supplementary materials

Michał Dudek[1,*], Grzegorz Siudem[2], Grzegorz Kwaśnik[3,4], Wojciech Żołnowski[3], and Marek T. Życzkowski[3]

[1]Military University of Technology, Institute of Applied Physics, Warsaw, 00908, Poland
[2]Warsaw University of Technology, Faculty of Physics, Warsaw, 00662, Poland
[3]Military University of Technology, Institute of Optoelectronics, Warsaw, 00908, Poland
[4]National Center for Nuclear Research, Department of Complex Systems, Otwock, 05400, Poland
[*]michal.dudek@wat.edu.pl

ABSTRACT

Supplementary materials for *Optical fibre-based quantum random number generator — stochastic modelling and measurements*.

Problem setup

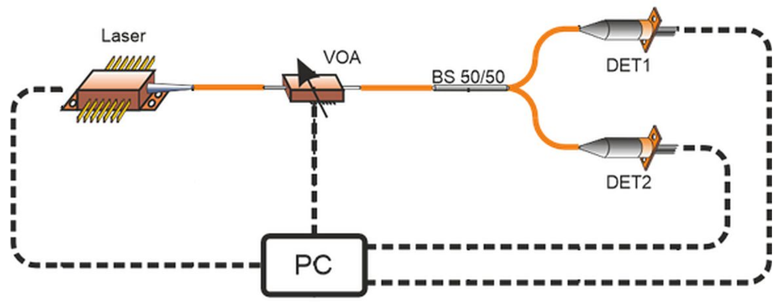

Figure 1. Experimental setup scheme.

Notation description:

- Random variable $\mathscr{N}$ describing the number of photons in the laser beam given from Poisson Distribution with parameter λ i.e., $\mathbb{P}(\mathscr{N}=N)=\frac{\lambda^N e^{-\lambda}}{N!}$.
- $\mathscr{N}_1$ and $\mathscr{N}_2$ are random variables describing the number of photons reaching the first and second detectors, respectively.
- $\mathscr{M}$ is a random variable describing the number of reflected or attenuated photons (photons lost in the system).
- We assume conservation of the number of photons $\mathscr{N}=\mathscr{N}_1+\mathscr{N}_2+\mathscr{M}$ and their probability mass function given with

$$\mathbb{P}(\mathscr{N}_1=n_1\wedge\mathscr{N}_2=n_2\wedge\mathscr{M}=m)=\frac{(n_1+n_2+m)!}{n_1!n_2!m!}p_1^{n_1}p_2^{n_2}s^m,$$

  for brevity with the notion of the multinomial symbol

$$\mathbb{P}(\mathscr{N}_1=n_1\wedge\mathscr{N}_2=n_2\wedge\mathscr{M}=m)=\binom{N}{n_1,n_2,m}p_1^{n_1}p_2^{n_2}s^m,$$

  where p_1, p_2, $s\in(0,1)$ are probabilities that photon reached detector 1, 2 or was lost in the system, respectively, and $p_1+p_2+s=1$.

- The reading of the ℓth detector at time t is given by the random variable $\Gamma_t^{(\ell)}$, which takes the values 0 or 1.
- The two-dimensional random variable $\Sigma_t = (\Gamma_t^{(1)}, \Gamma_t^{(2)})$ describes the state of both detectors at time t.

Derivation

Let us start with the definition of the series of random variables $\Sigma_t = \left(\Gamma_t^{(1)}, \Gamma_t^{(2)}\right)$ for fixed time step t and expand it with random variables $\mathcal{N} = \mathcal{N}_1 + \mathcal{N}_2 + \mathcal{M}$ conditioning over all possible configurations $n_1 + n_2 + m = N$, where n_ℓ is the number of photons arriving at ℓth detector, m is the number of photons lost in the system (attenuated or reflected) and N is the total number of photons introduced into the system. For $\sigma_\ell \in \{0,1\}$ one gets

$$
\begin{aligned}
&\mathbb{P}\left[\Sigma_t = (\sigma_1,\sigma_2)\right] = \mathbb{P}\left(\Gamma_t^{(1)} = \sigma_1 \wedge \Gamma_t^{(2)} = \sigma_2\right) = \\
&= \sum_{N=0}^{\infty} \mathbb{P}(\mathcal{N} = N)\mathbb{P}\left(\Gamma_t^{(1)} = \sigma_1 \wedge \Gamma_t^{(2)} = \sigma_2 \middle| \mathcal{N}_1 + \mathcal{N}_2 + \mathcal{M} = N\right) = \\
&= \sum_{N=0}^{\infty} \mathbb{P}(\mathcal{N} = N) \sum_{\substack{n_1,n_2,m\geqslant 0\\ n_1+n_2+m=N}} \mathbb{P}\left(\Gamma_t^{(1)} = \sigma_1 \wedge \Gamma_t^{(2)} = \sigma_2 \middle| \mathcal{N}_1 = n_1 \wedge \mathcal{N}_2 = n_2 \wedge \mathcal{M} = m\right) \times \\
&\quad \times \mathbb{P}\left(\mathcal{N}_1 = n_1 \wedge \mathcal{N}_2 = n_2 \wedge \mathcal{M} = m\right) = (\star),
\end{aligned}
$$

where in $(\star)$ we take advantage of the independence

$$
\begin{aligned}
&\mathbb{P}\left(\Gamma_t^{(1)} = \sigma_1 \wedge \Gamma_t^{(2)} = \sigma_2 \middle| \mathcal{N}_1 = n_1 \wedge \mathcal{N}_2 = n_2 \wedge \mathcal{M} = m\right) = \\
&= \mathbb{P}\left(\Gamma_t^{(1)} = \sigma_1 \middle| \mathcal{N}_1 = n_1\right) \times \mathbb{P}\left(\Gamma_t^{(2)} = \sigma_2 \middle| \mathcal{N}_2 = n_2\right),
\end{aligned}
$$

which leads to, where we know the probability mass function for $\mathcal{N}$ and $(\mathcal{N}_1, \mathcal{N}_2, \mathcal{M})$

$$
\begin{aligned}
(\star) &= \sum_{N=0}^{\infty} \mathbb{P}(\mathcal{N} = N) \sum_{\substack{n_1,n_2,m\geqslant 0\\ n_1+n_2+m=N}} \mathbb{P}\left(\Gamma_t^{(1)} = \sigma_1 \middle| \mathcal{N}_1 = n_1\right) \times \mathbb{P}\left(\Gamma_t^{(2)} = \sigma_2 \middle| \mathcal{N}_2 = n_2\right) \times \\
&\quad \times \mathbb{P}\left(\mathcal{N}_1 = n_1 \wedge \mathcal{N}_2 = n_2 \wedge \mathcal{M} = m\right) = \\
&= \sum_{N=0}^{\infty} \frac{\lambda^N}{N!} e^{-\lambda} \Bigg\{ \mathbb{P}\left(\Gamma_t^{(1)} = \sigma_1 \middle| \mathcal{N}_1 = 0\right) \mathbb{P}\left(\Gamma_t^{(2)} = \sigma_2 \middle| \mathcal{N}_2 = 0\right) \binom{N}{0,0,N} s^N + \\
&\quad + \sum_{m=0}^{N-1} \Bigg[\mathbb{P}\left(\Gamma_t^{(1)} = \sigma_1 \middle| \mathcal{N}_1 = 0\right) \mathbb{P}\left(\Gamma_t^{(2)} = \sigma_2 \middle| \mathcal{N}_2 = N-m\right) \binom{N}{0,N-m,m} p_2^{N-m} s^m + \\
&\quad + \mathbb{P}\left(\Gamma_t^{(1)} = \sigma_1 \middle| \mathcal{N}_1 = N-m\right) \mathbb{P}\left(\Gamma_t^{(2)} = \sigma_2 \middle| \mathcal{N}_2 = 0\right) \binom{N}{N-m,0,m} p_1^{N-m} s^m + \\
&\quad + \sum_{\substack{n_1,n_2>0\\ n_1+n_2=N-m}} \left(\mathbb{P}\left(\Gamma_t^{(1)} = \sigma_1 \middle| \mathcal{N}_1 = n_1\right) \mathbb{P}\left(\Gamma_t^{(2)} = \sigma_2 \middle| \mathcal{N}_2 = n_2\right) \binom{N}{n_1,n_2,m} p_1^{n_1} p_2^{n_2} s^m \right) \Bigg] \Bigg\} = \\
&= \mathbb{P}\left(\Gamma_t^{(1)} = \sigma_1 \middle| \mathcal{N}_1 = 0\right) \mathbb{P}\left(\Gamma_t^{(2)} = \sigma_2 \middle| \mathcal{N}_2 = 0\right) \sum_{N=0}^{\infty} \frac{\lambda^N s^N}{N!} e^{-\lambda} + \\
&\quad + \sum_{N=0}^{\infty} \frac{\lambda^N}{N!} e^{-\lambda} \sum_{m=0}^{N-1} \Bigg[\mathbb{P}\left(\Gamma_t^{(1)} = \sigma_1 \middle| \mathcal{N}_1 = 0\right) \mathbb{P}\left(\Gamma_t^{(2)} = \sigma_2 \middle| \mathcal{N}_2 = N-m\right) \binom{N}{m} p_2^{N-m} s^m + \\
&\quad + \mathbb{P}\left(\Gamma_t^{(1)} = \sigma_1 \middle| \mathcal{N}_1 = N-m\right) \mathbb{P}\left(\Gamma_t^{(2)} = \sigma_2 \middle| \mathcal{N}_2 = 0\right) \binom{N}{m} p_1^{N-m} s^m + \\
&\quad + \sum_{\substack{n_1,n_2>0\\ n_1+n_2=N-m}} \left(\mathbb{P}\left(\Gamma_t^{(1)} = \sigma_1 \middle| \mathcal{N}_1 = n_1\right) \mathbb{P}\left(\Gamma_t^{(2)} = \sigma_2 \middle| \mathcal{N}_2 = n_2\right) \binom{N}{n_1,n_2,m} p_1^{n_1} p_2^{n_2} s^m \right) \Bigg] \Bigg\} = (\star\star).
\end{aligned}
$$

We stop further derivation at this point and focus on the exact formula of the probabilities $\mathbb{P}\left(\Gamma_t^{(i)}=\sigma\middle|\mathcal{N}_i=n_i\right)$, which describe detection properties of both $i=1,2$ detectors

$$\mathbb{P}\left(\Gamma_t^{(i)}=\sigma\middle|\mathcal{N}_i=n\right)=\sigma+(1-2\sigma)(1-q_i)^n \text{ for } n>0,$$
$$\mathbb{P}\left(\Gamma_t^{(i)}=\sigma\middle|\mathcal{N}_i=0\right)=\sigma+(1-2\sigma)r_i,$$

where

- $r_i\in(0,\,1)$ describes the probability that the detector will not detect any photons when there are no photons to be detected (true negative),
- $q_i\in(0,\,1)$ describes the probability that the detector will detect photons when there are photons to be detected (true positive) – in other words, it represents the photon detection efficiency.

This allows to further transform $(\star\star)$ as follows

$$\begin{aligned}
(\star\star)=&[\sigma_1+(1-2\sigma_1)r_1][\sigma_2+(1-2\sigma_2)r_2]e^{-\lambda}\sum_{N=0}^{\infty}\frac{\lambda^N s^N}{N!}+\\
&+[\sigma_1+(1-2\sigma_1)r_1]e^{-\lambda}\sum_{N=0}^{\infty}\frac{\lambda^N}{N!}\sum_{m=0}^{N-1}\left[\sigma_2+(1-2\sigma_2)(1-q_2)^{N-m}\right]\binom{N}{m}p_2^{N-m}s^m+\\
&+[\sigma_2+(1-2\sigma_2)r_2]e^{-\lambda}\sum_{N=0}^{\infty}\frac{\lambda^N}{N!}\sum_{m=0}^{N-1}\left[\sigma_1+(1-2\sigma_1)(1-q_1)^{N-m}\right]\binom{N}{m}p_1^{N-m}s^m+\\
&+\sum_{N=0}^{\infty}\frac{\lambda^N e^{-\lambda}}{N!}\sum_{m=0}^{N-1}\sum_{\substack{n_1,n_2>0\\ n_1+n_2=N-m}}[\sigma_1+(1-2\sigma_1)(1-q_1)^{n_1}][\sigma_2+(1-2\sigma_2)(1-q_2)^{n_2}]\times\binom{N}{n_1,n_2,m}p_1^{n_1}p_2^{n_2}s^m=\\
=&[\sigma_1+(1-2\sigma_1)r_1][\sigma_2+(1-2\sigma_2)r_2]e^{-\lambda}\sum_{N=0}^{\infty}\frac{\lambda^N s^N}{N!}+\\
&+[\sigma_1+(1-2\sigma_1)r_1]e^{-\lambda}\sum_{N=0}^{\infty}\frac{\lambda^N}{N!}\left[\sigma_2\sum_{m=0}^{N-1}\binom{N}{m}p_2^{N-m}s^m+(1-2\sigma_2)\sum_{m=0}^{N-1}\binom{N}{m}[p_2(1-q_2)]^{N-m}s^m\right]+\\
&+[\sigma_2+(1-2\sigma_2)r_2]e^{-\lambda}\sum_{N=0}^{\infty}\frac{\lambda^N}{N!}\left[\sigma_1\sum_{m=0}^{N-1}\binom{N}{m}p_1^{N-m}s^m+(1-2\sigma_1)\sum_{m=0}^{N-1}\binom{N}{m}[p_1(1-q_1)]^{N-m}s^m\right]+\\
&+\sum_{N=0}^{\infty}\frac{\lambda^N e^{-\lambda}}{N!}\sum_{m=0}^{N-1}\sum_{\substack{n_1,n_2>0\\ n_1+n_2=N-m}}\binom{N}{n_1,n_2,m}\Bigg[\sigma_1\sigma_2p_1^{n_1}p_2^{n_2}s^m+\sigma_2(1-2\sigma_1)[p_1(1-q_1)]^{n_1}p_2^{n_2}s^m+\\
&+\sigma_1(1-2\sigma_2)p_1^{n_1}[p_2(1-q_2)]^{n_2}s^m+(1-2\sigma_1)(1-2\sigma_2)[p_1(1-q_1)]^{n_1}[p_2(1-q_2)]^{n_2}s^m\Bigg]=(\star\star\star).
\end{aligned}$$

Let us recall the binomial theorem

$$\sum_{m=0}^{N}\binom{N}{m}a^{N-m}b^m=(a+b)^N,$$

and the multinomial theorem

$$\sum_{\substack{n_1,n_2,n_3\geqslant 0\\ n_1+n_2+n_3=N}}\binom{N}{n_1,n_2,n_3}a^{n_1}b^{n_2}c^{n_3}=(a+b+c)^N,$$

with their notion one can simplify $(\star\star\star)$ in the following way (for clarity under the last sum we use abbreviation instead of the whole term)

$$
\begin{aligned}
(\star\star\star) = {} & [\sigma_1 + (1-2\sigma_1) r_1]\,[\sigma_2 + (1-2\sigma_2) r_2]\, e^{-\lambda} \sum_{N=0}^{\infty} \frac{\lambda^N s^N}{N!} + \\
& + [\sigma_1 + (1-2\sigma_1) r_1] \sum_{N=0}^{\infty} \frac{e^{-\lambda}\lambda^N}{N!} \left[\sigma_2 \left(\sum_{m=0}^{N} \binom{N}{m} p_2^{N-m} s^m - s^N \right) + \right. \\
& \quad \left. + (1-2\sigma_2) \left(\sum_{m=0}^{N} \binom{N}{m} [p_2(1-q_2)]^{N-m} s^m - s^N \right) \right] + \\
& + [\sigma_2 + (1-2\sigma_2) r_2] \sum_{N=0}^{\infty} \frac{e^{-\lambda}\lambda^N}{N!} \left[\sigma_1 \left(\sum_{m=0}^{N} \binom{N}{m} p_1^{N-m} s^m - s^N \right) + \right. \\
& \quad \left. + (1-2\sigma_1) \left(\sum_{m=0}^{N} \binom{N}{m} [p_1(1-q_1)]^{N-m} s^m - s^N \right) \right] + \\
& + \sum_{N=0}^{\infty} \frac{\lambda^N e^{-\lambda}}{N!} \left[\sum_{\substack{n_1, n_2, m \geqslant 0 \\ n_1+n_2+m=N}} \Big(\cdots \Big)_{(n_1,n_2,m)} - \sum_{n_1=0}^{N} \Big(\cdots \Big)_{(n_1,0,N-n_1)} + \right. \\
& \quad \left. - \sum_{n_2=0}^{N} \Big(\cdots \Big)_{(0,n_2,N-n_2)} + \Big(\cdots \Big)_{(0,0,N)} \right]
\end{aligned}
$$

$$(\star\star\star) = [\sigma_1 + (1-2\sigma_1)r_1]\,[\sigma_2 + (1-2\sigma_2)r_2]\,e^{-\lambda}\sum_{N=0}^{\infty}\frac{\lambda^N s^N}{N!} +$$

$$+ [\sigma_1 + (1-2\sigma_1)r_1]\,e^{-\lambda}\sum_{N=0}^{\infty}\frac{\lambda^N}{N!}\left\{\sigma_2\left[(p_2+s)^N - s^N\right] + (1-2\sigma_2)\left[(p_2(1-q_2)+s)^N - s^N\right]\right\} +$$

$$+ [\sigma_2 + (1-2\sigma_2)r_2]\,e^{-\lambda}\sum_{N=0}^{\infty}\frac{\lambda^N}{N!}\left\{\sigma_1\left[(p_1+s)^N - s^N\right] + (1-2\sigma_1)\left[(p_1(1-q_1)+s)^N - s^N\right]\right\} +$$

$$+ \sum_{N=0}^{\infty}\frac{\lambda^N e^{-\lambda}}{N!}\Bigg[\Bigg(\sigma_1\sigma_2(p_1+p_2+s)^N + \sigma_2(1-2\sigma_1)\,[p_1(1-q_1)+p_2+s]^N +$$

$$+ \sigma_1(1-2\sigma_2)\,[p_1+p_2(1-q_2)+s]^N + (1-2\sigma_1)(1-2\sigma_2)\,[p_1(1-q_1)+p_2(1-q_2)+s]^N\Bigg) +$$

$$- \Bigg(\sigma_1\sigma_2(p_1+s)^N + \sigma_2(1-2\sigma_1)\,[p_1(1-q_1)+s]^N + \sigma_1(1-2\sigma_2)(p_1+s)^N +$$

$$+ (1-2\sigma_1)(1-2\sigma_2)\,[p_1(1-q_1)+s]^N\Bigg) - \Bigg(\sigma_1\sigma_2(p_2+s)^N + \sigma_2(1-2\sigma_1)(p_2+s)^N +$$

$$+ \sigma_1(1-2\sigma_2)\,[p_2(1-q_2)+s]^N + (1-2\sigma_1)(1-2\sigma_2)\,[p_2(1-q_2)+s]^N\Bigg) +$$

$$+ \Bigg(\sigma_1\sigma_2 + \sigma_2(1-2\sigma_1) + \sigma_1(1-2\sigma_2) + (1-2\sigma_1)(1-2\sigma_2)\Bigg)s^N\Bigg] =$$

$$= [\sigma_1 + (1-2\sigma_1)r_1]\,[\sigma_2 + (1-2\sigma_2)r_2]\,e^{-\lambda}\sum_{N=0}^{\infty}\frac{\lambda^N s^N}{N!} +$$

$$+ [\sigma_1 + (1-2\sigma_1)r_1]\,e^{-\lambda}\sum_{N=0}^{\infty}\frac{\lambda^N}{N!}\left[\sigma_2(p_2+s)^N + (1-2\sigma_2)(p_2(1-q_2)+s)^N - (1-\sigma_2)s^N\right] +$$

$$+ [\sigma_2 + (1-2\sigma_2)r_2]\,e^{-\lambda}\sum_{N=0}^{\infty}\frac{\lambda^N}{N!}\left[\sigma_1(p_1+s)^N + (1-2\sigma_1)(p_1(1-q_1)+s)^N - (1-\sigma_1)s^N\right] +$$

$$+ \sum_{N=0}^{\infty}\frac{\lambda^N e^{-\lambda}}{N!}\Bigg[\Bigg(\sigma_1\sigma_2(p_1+p_2+s)^N + \sigma_2(1-2\sigma_1)\,[p_1(1-q_1)+p_2+s]^N +$$

$$+ \sigma_1(1-2\sigma_2)\,[p_1+p_2(1-q_2)+s]^N + (1-2\sigma_1)(1-2\sigma_2)\,[p_1(1-q_1)+p_2(1-q_2)+s]^N\Bigg) +$$

$$- \Bigg(\sigma_1(1-\sigma_2)(p_1+s)^N + (1-2\sigma_1)(1-\sigma_2)\,[p_1(1-q_1)+s]^N\Bigg) +$$

$$- \Bigg(\sigma_2(1-\sigma_1)(p_2+s)^N + (1-\sigma_1)(1-2\sigma_2)\,[p_2(1-q_2)+s]^N\Bigg) + (1-\sigma_1)(1-\sigma_2)s^N\Bigg] = (\spadesuit).$$

Let us recall the well-known Poissonian summation rule

$$\sum_{N=0}^{\infty}\frac{\lambda^N e^{-\lambda}}{N!}\phi^n = e^{(\phi-1)\lambda},$$

which leads to the further simplifications of $(\spadesuit)$

$$(\spadesuit) = [\sigma_1 + (1-2\sigma_1)r_1]\,[\sigma_2 + (1-2\sigma_2)r_2]\,e^{(s-1)\lambda} +$$
$$+ [\sigma_1 + (1-2\sigma_1)r_1]\left[\sigma_2 e^{(p_2+s-1)\lambda} + (1-2\sigma_2)e^{[p_2(1-q_2)+s-1]\lambda} - (1-\sigma_2)e^{(s-1)\lambda}\right] +$$
$$+ [\sigma_2 + (1-2\sigma_2)r_2]\left[\sigma_1 e^{(p_1+s-1)\lambda} + (1-2\sigma_1)e^{[p_1(1-q_1)+s-1)]\lambda} - (1-\sigma_1)e^{(s-1)\lambda}\right] +$$
$$+ \Bigg[\Bigg(\sigma_1\sigma_2 e^{(p_1+p_2+s-1)\lambda} + \sigma_2(1-2\sigma_1)e^{[p_1(1-q_1)+p_2+s-1]\lambda} +$$
$$+ \sigma_1(1-2\sigma_2)e^{[p_1+p_2(1-q_2)+s-1]\lambda} + (1-2\sigma_1)(1-2\sigma_2)e^{[p_1(1-q_1)+p_2(1-q_2)+s-1]\lambda}\Bigg) +$$
$$- \Bigg(\sigma_1(1-\sigma_2)e^{(p_1+s-1)\lambda} + (1-2\sigma_1)(1-\sigma_2)e^{[p_1(1-q_1)+s-1]\lambda}\Bigg) +$$
$$- \Bigg(\sigma_2(1-\sigma_1)e^{(p_2+s-1)\lambda} + (1-\sigma_1)(1-2\sigma_2)e^{[p_2(1-q_2)+s-1]\lambda}\Bigg) +$$
$$+ (1-\sigma_1)(1-\sigma_2)e^{(s-1)\lambda}\Bigg] =$$
$$= e^{(s-1)\lambda}\Big[(1-r_1)(1-r_2)\underbrace{(1-2\sigma_1)}_{=(-1)^{\sigma_1}}\underbrace{(1-2\sigma_2)}_{=(-1)^{\sigma_2}} + e^{p_1\lambda}\sigma_1(1-r_2)(\underbrace{2\sigma_2-1}_{=-(-1)^{\sigma_2}}) +$$
$$+ e^{p_2\lambda}\sigma_2(1-r_1)(\underbrace{2\sigma_1-1}_{=-(-1)^{\sigma_1}}) + e^{p_1(1-q_1)\lambda}(1-r_2)\underbrace{(1-2\sigma_1)}_{=(-1)^{\sigma_1}}(\underbrace{2\sigma_2-1}_{=-(-1)^{\sigma_2}}) +$$
$$+ e^{p_2(1-q_2)\lambda}(1-r_1)\underbrace{(1-2\sigma_1)}_{=(-1)^{\sigma_1}}(\underbrace{2\sigma_2-1}_{=-(-1)^{\sigma_2}}) + e^{(p_1+p_2)\lambda}\sigma_1\sigma_2 + e^{[p_1(1-q_1)+p_2]\lambda}\sigma_2\underbrace{(1-2\sigma_1)}_{=(-1)^{\sigma_1}} +$$
$$+ e^{[p_1+p_2(1-q_2)]\lambda}\sigma_1\underbrace{(1-2\sigma_2)}_{=(-1)^{\sigma_2}} + e^{[p_1(1-q_1)+p_2(1-q_2)]\lambda}\underbrace{(1-2\sigma_1)}_{=(-1)^{\sigma_1}}\underbrace{(1-2\sigma_2)}_{=(-1)^{\sigma_2}}\Big] =$$
$$= e^{(s-1)\lambda}\Big[(1-r_1)(1-r_2)(-1)^{\sigma_1+\sigma_2} - e^{p_1\lambda}\sigma_1(1-r_2)(-1)^{\sigma_2} - e^{p_2\lambda}\sigma_2(1-r_1)(-1)^{\sigma_1} +$$
$$- e^{p_1(1-q_1)\lambda}(1-r_2)(-1)^{\sigma_1+\sigma_2} - e^{p_2(1-q_2)\lambda}(1-r_1)(-1)^{\sigma_1+\sigma_2} + e^{(p_1+p_2)\lambda}\sigma_1\sigma_2 +$$
$$+ e^{[p_1(1-q_1)+p_2]\lambda}\sigma_2(-1)^{\sigma_1} + e^{[p_1+p_2(1-q_2)]\lambda}\sigma_1(-1)^{\sigma_2} + e^{[p_1(1-q_1)+p_2(1-q_2)]\lambda}(-1)^{\sigma_1+\sigma_2}\Big],$$

which could be finally simplified in the following form

$$\mathbb{P}\left[\Sigma_t = (\sigma_1,\sigma_2)\right] = e^{(s-p_1q_1-p_2q_2-1)\lambda}(-1)^{\sigma_1+\sigma_2}\left[(1-r_1)e^{\lambda p_1q_1} + \sigma_1(-1)^{\sigma_1}e^{\lambda p_1(q_1+1)} + e^{\lambda p_1}\right] \times$$
$$\times \left[(1-r_2)e^{\lambda p_2q_2} + \sigma_2(-1)^{\sigma_2}e^{\lambda p_2(q_2+1)} + e^{\lambda p_2}\right].$$

Finally, we change $p_1 := p_1 - s_1$ and $p_2 := p_2 - s_2$, where s_i are loss factors in both optical fibre paths and so far we have not distinguished their source for simplicity's sake. The values p_1 and p_2 without the influence of s_1 and s_2 may be considered probabilities of finding photon in the path either 1 or 2, respectively, i.e., parameters of the optical fibre splitter (splitting ratio), while s_1 and s_2 are probabilities of photons being reflected or attenuated in path 1 or 2, respectively. Of course, when we discard losses in these two paths ($s_1 = 0$ and $s_2 = 0$), the probability that the photon reaches a detector is the same as the probability of finding a photon in its path. Hence, the final probability of the photon loss in the system can be written as $s = 1-(p_1-s_1)-(p_2-s_2)$. This leads to the final formula

$$\mathbb{P}\left[\Sigma_t = (\sigma_1,\sigma_2)\right] = e^{-\lambda[(p_1-s_1)(q_1+1)+(p_2-s_2)(q_2+1)]}(-1)^{\sigma_1+\sigma_2} \times$$
$$\times \left[(1-r_1)e^{\lambda(p_1-s_1)q_1} + \sigma_1(-1)^{\sigma_1}e^{\lambda(p_1-s_1)(q_1+1)} + e^{\lambda(p_1-s_1)}\right] \times$$
$$\times \left[(1-r_2)e^{\lambda(p_2-s_2)q_2} + \sigma_2(-1)^{\sigma_2}e^{\lambda(p_2-s_2)(q_2+1)} + e^{\lambda(p_2-s_2)}\right].$$

The next step in our derivation is the differentiation of the detectors properties regarding their previous states. i.e., for $i = 1, 2$ we use

- r_i, q_i when the previous measurement was negative: $\sigma_i(t-1) = 0$,
- r_i', q_i' when the previous measurement was positive: $\sigma_i(t-1) = 1$.

Parameters with a prime sign differ from parameters without this sign due to the afterpulsing phenomenon, which we model with parameters Δ_1, Δ_2

$$r_i' = r_i - \Delta_i, \quad q_i' = q_i + \Delta_i.$$

The above allows one to compute the transition matrix $\mathscr{A}$ which governs the changes of the probability i.e., (for brevity we skip brackets, e.g., $(0,0) = 00$)

$$\begin{pmatrix} \mathbb{P}[\Sigma_t = 00] \\ \mathbb{P}[\Sigma_t = 01] \\ \mathbb{P}[\Sigma_t = 10] \\ \mathbb{P}[\Sigma_t = 11] \end{pmatrix} = \mathscr{A} \begin{pmatrix} \mathbb{P}[\Sigma_{t-1} = 00] \\ \mathbb{P}[\Sigma_{t-1} = 01] \\ \mathbb{P}[\Sigma_{t-1} = 10] \\ \mathbb{P}[\Sigma_{t-1} = 11] \end{pmatrix},$$

where $\mathscr{A}$ is defined as follows

$$\mathscr{A} = \begin{pmatrix} \mathbb{P}[\Sigma_t = 00|\Sigma_{t-1} = 00] & \mathbb{P}[\Sigma_t = 01|\Sigma_{t-1} = 00] & \mathbb{P}[\Sigma_t = 10|\Sigma_{t-1} = 00] & \mathbb{P}[\Sigma_t = 11|\Sigma_{t-1} = 00] \\ \mathbb{P}[\Sigma_t = 00|\Sigma_{t-1} = 01] & \mathbb{P}[\Sigma_t = 01|\Sigma_{t-1} = 01] & \mathbb{P}[\Sigma_t = 10|\Sigma_{t-1} = 01] & \mathbb{P}[\Sigma_t = 11|\Sigma_{t-1} = 01] \\ \mathbb{P}[\Sigma_t = 00|\Sigma_{t-1} = 10] & \mathbb{P}[\Sigma_t = 01|\Sigma_{t-1} = 10] & \mathbb{P}[\Sigma_t = 10|\Sigma_{t-1} = 10] & \mathbb{P}[\Sigma_t = 11|\Sigma_{t-1} = 10] \\ \mathbb{P}[\Sigma_t = 00|\Sigma_{t-1} = 11] & \mathbb{P}[\Sigma_t = 01|\Sigma_{t-1} = 11] & \mathbb{P}[\Sigma_t = 10|\Sigma_{t-1} = 11] & \mathbb{P}[\Sigma_t = 11|\Sigma_{t-1} = 11] \end{pmatrix}.$$

We are interested in the ergodic distribution for a Markov process given with matrix $\mathscr{A}$. In other words, we want to find the limit value

$$\begin{pmatrix} \pi_{00} \\ \pi_{01} \\ \pi_{10} \\ \pi_{11} \end{pmatrix} = \lim_{t\to\infty} \begin{pmatrix} \mathbb{P}[\Sigma_t = 00] \\ \mathbb{P}[\Sigma_t = 01] \\ \mathbb{P}[\Sigma_t = 10] \\ \mathbb{P}[\Sigma_t = 11] \end{pmatrix}, \tag{1}$$

which is given as eigenvector of $\mathscr{A}$ with eigenvalue 1, i.e.,

$$\begin{pmatrix} \pi_{00} \\ \pi_{01} \\ \pi_{10} \\ \pi_{11} \end{pmatrix} = \mathscr{A} \begin{pmatrix} \pi_{00} \\ \pi_{01} \\ \pi_{10} \\ \pi_{11} \end{pmatrix}.$$

The above eigenproblem, together with the normalization condition $\pi_{00} + \pi_{01} + \pi_{10} + \pi_{11} = 1$ leads to

$$\begin{aligned}
\pi_{00} =& C\left[e^{\lambda(p_1-s_1)} + (r_1-\Delta_1-1)\,e^{\lambda(p_1-s_1)(q_1+\Delta_1)}\right]\left[e^{\lambda(p_2-s_2)} + (r_2-\Delta_2-1)\,e^{\lambda(p_2-s_2)(q_2+\Delta_2)}\right],\\
\pi_{01} =& Ce^{\lambda[\Delta_2(p_2-s_2)+p_1-s_1]}\left[e^{\lambda(p_2-s_2)} - e^{\lambda(p_2-s_2)(q_2+1)} + (r_2-1)\,e^{\lambda(p_2-s_2)q_2}\right]\times\\
&\times\left[(-r_1+\Delta_1+1)\,e^{\lambda(p_1-s_1)(q_1+\Delta_1-1)} - 1\right],\\
\pi_{10} =& Ce^{\lambda[\Delta_1(p_1-s_1)+p_2-s_2]}\left[e^{\lambda(p_1-s_1)} - e^{\lambda(p_1-s_1)(q_1+1)} + (r_1-1)\,e^{\lambda(p_1-s_1)q_1}\right]\times\\
&\times\left[(-r_2+\Delta_2+1)\,e^{\lambda(p_2-s_2)(q_2+\Delta_2-1)} - 1\right],\\
\pi_{11} =& Ce^{\lambda[\Delta_1(p_1-s_1)+\Delta_2(p_2-s_2)]}\left[e^{\lambda(p_1-s_1)} - e^{\lambda(p_1-s_1)(q_1+1)} + (r_1-1)\,e^{\lambda(p_1-s_1)q_1}\right]\times\\
&\left[e^{\lambda(p_2-s_2)} - e^{\lambda(p_2-s_2)(q_2+1)} + (r_2-1)\,e^{\lambda(p_2-s_2)q_2}\right],\\
C =& \left[-e^{(\Delta_1+1)\lambda(p_1-s_1)} + e^{\lambda(p_1-s_1)} + \Delta_1\left(-e^{\lambda(p_1-s_1)(q_1+\Delta_1)}\right) + e^{\lambda(p_1-s_1)(q_1+\Delta_1+1)}\right]^{-1}\times\\
&\left[-e^{(\Delta_2+1)\lambda(p_2-s_2)} + e^{\lambda(p_2-s_2)} + \Delta_2\left(-e^{\lambda(p_2-s_2)(q_2+\Delta_2)}\right) + e^{\lambda(p_2-s_2)(q_2+\Delta_2+1)}\right]^{-1}.
\end{aligned}$$
